# Biophysical Characteristics of Meridians and Acupoints: A Systematic Review

**DOI:** 10.1155/2012/793841

**Published:** 2012-12-30

**Authors:** Juan Li, Qing Wang, Huiling Liang, Haoxu Dong, Yan Li, Ernest Hung Yu Ng, Xiaoke Wu

**Affiliations:** ^1^Department of Obstetrics and Gynecology, National Key Discipline and Clinical Base, Heilongjiang University of Chinese Medicine, 150040 Harbin, China; ^2^Department of Obstetrics & Gynecology, The University of Hong Kong, Hong Kong

## Abstract

As an integral part of traditional Chinese medicine (TCM), acupuncture is a convenient and effective therapy with fewer adverse effects. Recently, researches on meridian essence have become core issues of modern TCM. Numerous experiments have demonstrated the objective existence of meridians by different technologies since 1950s, such as biophysics, biochemistry, and molecular biology. In this paper, we review biophysical studies on electric, acoustic, thermal, optical, magnetic, isotopic, and myoelectric aspects of meridians and acupoints. These studies suggest that meridians/acupoints have biophysical characteristics which are different from nonacupuncture points. Owing to the limitations of previous studies, future research using high-throughput technologies such as omics and multicenter randomized controlled trials should be performed to explore the acupuncture's mechanisms of action and demonstration of efficacy.

## 1. Introduction

Acupuncture is an integral part of traditional Chinese medicine (TCM), which dates back about 3,000 years [1]. It is increasingly being accepted as a complementary and alternative medicine (CAM) therapy in the United States [2]. The practice of acupuncture is based on the meridian system [3], and along the meridians lie acupuncture points or acupoints, which may have therapeutic effects for certain medical conditions when stimulated by needling, pressure, or heat [3].

The meridian theory is a fundamental part of TCM and has guided acupuncture for thousands of years [4]. While the clinical effects of acupuncture have drawn extensive attentions from researchers of science and medicine, lots of questions arise: Do meridians exist? What are the mechanisms of their actions? What is the physiological basis of meridians? What are the differences between acupoints and nonacupuncture points? So far, these questions have not been completely answered, but a large number of experiments and clinical trials have been conducted using advanced biophysical, biochemical, and molecular biological methods [5–8].

In order to have a better understanding of the biophysical basis of meridians/acupoints and their mechanisms of action, we review the biophysical studies of meridians/acupoints after searching the electronic databases MEDLINE, EMBASE, CINAHL, EMBR, CNKI, Wanfang, and Vip from the beginning to June 2012.

## 2. Biophysical Characteristics of Meridians and Acupoints

### 2.1. Electrical Characteristics

Low resistance and high capacitance are generally accepted as electrical characteristics of meridians and acupoints [9–11]. Currently, researchers are evaluating the electrophysiological properties of acupoints as a possible means to explore the acting mechanisms of acupuncture [12]. Wang et al. [13] used the infrared thermal images and surface resistance measurement to detect the temperature and resistance at left and right pericardium (PC) 6, right bladder (BL) 15, and points 1 cm away from all above acupoints as controls in 20 rabbits. The results showed that acupoints had high-temperature and low-resistance characteristics compared with nonacupuncture point controls, and the resistance values were closely related with skin temperature. Other researchers in China [14, 15] have also demonstrated this phenomenon. In addition, Egot-Lemaire and Ziskin [16] found that the dielectric properties of the acupoints were somewhat different from those of the surrounding nonacupuncture points, which ranged from 50 GHz to about 61 GHz.

However, the above findings are not consistent with results of other studies. Ahn et al. [17] used a four-electrode method to measure the electrical impedance along segments of the pericardium and spleen meridians and corresponding parallel control segments in 23 human subjects. The results showed that tissue impedance was on average lower along the pericardium meridian, but not along the spleen meridian, compared with their respective controls. Pearson et al. [18] used two instruments to test the electrical skin impedance at three acupoints and their nearby sites. The results showed that none of the three acupoints tested had lower skin impedance than at either of the nearby control points. Ahn et al. [19] in a systematic review of studies on the electrical characteristics of acupuncture structures found only 5 out of 9 point studies showed positive association between acupoints and lower electrical resistance and impedance, while 7 out of 9 meridian studies showed positive association between acupuncture meridians and lower electrical impedance and higher capacitance. The evidence did not conclusively support the claim that acupoints or meridians were electrically distinguishable. Kramer et al. [20] used an array consisting of 64 (8 × 8) electrodes to measure the skin resistance. The electrodes were located at corresponding acupoints, and the results of electrical skin resistance measurements (ESRMs) were compared to those of the surrounding electrodes.

Table 1 showed that the electrical skin resistance (ESR) at most acupoints (62.8%) had no significant difference when compared with that of the surrounding areas. This was partly in line with some previous high quality studies, which draw relatively negative conclusions to this topic. Therefore, it could not be concluded that ESRMs could be used for acupoint localization or diagnostic/therapeutic purposes. In this paper, they also found that the reproducibility of the array for measuring ESR was extremely high after 1 minute but was low after 1 hour and 1 week. The phenomenon was characterized by high short-term and low long-term reproducibility. One simple explanation might be a change of environmental conditions, including the factors of artifacts, wide ESR variation inter- and intra-individually, and ESR shift which may be caused by transepidermal water loss or skin hydration [21]. Changes in the thickness of stratum corneum layer (e.g., small abrasions) and the electrode size could also influence ESRMs. And they found that the factor affecting reliability of the results might be that they simply failed to measure the acupoint itself due to the distance of 8 mm between the centers of the electrodes.

Other researchers [22, 23] also found electrode material, size and shape, pressure exerted by the probe, duration of probe application, inclination of the probe tip on the skin, and variations in skin condition (dry/moist, thickness, and integrity of the stratum corneum) might be the potential confounders affecting the reproducibility and reliability of the device for measuring skin electrical current/resistance. To resolve the limitations, Colbert et al. [12] designed a continuous recording system, a fully automatic multichannel device, to record skin impedance at multiple acupoints simultaneously over 24 hours. By using it, they successfully detected that the skin impedance of acupoints was lower than the nearby nonacupuncture points. Moreover, Colbert et al. [24] developed an automated multichannel prototype system and recorded electrical resistance and capacitance at eight skin sites in 33 healthy participants for over 2 hours. The results showed that only the acupoints on the liver and spleen meridians had a lower resistance than their nearby sites (4 mm away), while other comparisons showed no significant differences.

In order to explore the mechanisms of electrical characteristics, Yang [25] analyzed various possible factors that may lead to low resistance. He concluded that a relative higher content of the interstitial fluid (tissue fluid) was the source of low resistance, and the histological essence of meridians was the bands with relative high content of interstitial fluid in loose connective tissues. Others believed that gap junctions might be the structural basis that determined the different skin resistances [26–28]. Tan [29] considered that, besides the inherent organizational factors, the concentration change of charged molecules in the corresponding environment was one of the most important variable factors influencing the resistance in different parts of the body. Furthermore, according to the bioelectric field with rich ion along meridians, he concluded that the resistance of meridians was certain to reduce when propagated sensation along channel (PSC) happened. In other reports [30–35], local tissue-released noradrenalin (NA), nitric oxide (NO), tumor-related factors, and mast cell-released histamine and serotonin (5-HT) were considered to be responsible for the electrical characteristics of meridians and acupoints.

### 2.2. Acoustic Characteristics

The characteristics of phonation and transmitting sound in meridians were first reported in 1980s and researchers have tried to confirm them in both animal experiments and human trials. Zhang et al. [36] detected 8 acupoints on the conception vessel, 11 acupoints on the governor vessel, and control points on both sides 2 mm apart from the acupoints mentioned above in 30 rabbits. The results showed that the sound wave's amplitude of acupoints was significantly higher than nonacupuncture points. Wei et al. [37] measured the intensity of sound wave and confirmed that the sound wave could be transmitted in the meridians and acupoints. Its conduction velocity was faster than that of soft tissue but was lower than bone conduction. Furthermore, a significant relationship was found between the tones and the meridians, which were described in the *Yellow Emperor's Internal Medicine *(*Huang Di Nei Jin)* (Table 2) [38]. 

The acoustic characteristics of acupoints are concluded as follows: (A) frequency is 2–15 Hz; (B) amplitude is 0.5–10 mV; (C) waveform is similar to sharp wave or sine wave; (D) bidirectional conduction velocity is 6.2–10 cm/s; (E) it could be blocked [39].

The acoustic conduction depends on the media, and orderly the distribution of microscopic particles in the media is a necessary condition to promote the effective conduction. It was reported that the property of transmitting sound in meridians is better than that of the surrounding areas due to the enrichment of isotropic ions along meridians under the action of bioelectric field [26]. Moreover, main and collateral channels are proved to be good medium for mechanical vibration wave and infrasonic wave [39, 40]. Perhaps this is caused by the characteristics of infrasonic wave, that is, long wavelength, weak attenuation extent, difficult to be absorbed while spreading, and strong penetration [40].

In addition, Krevsky et al. [41] found that acoustic characteristics of meridians and acupoints could be used for diagnostic purposes. Transmitted microwave along the meridians was able to detect on the next acupoint of the same meridian. The frequency of microwave in cancer patients was different from that of healthy controls. And the frequency ranges of the microwave transmitted in different meridians were found to be different.

### 2.3. Thermal Characteristics

Wang [42] reviewed that Borsarello found the trace of facial isotherm detected by the infrared thermal images was similar with human meridians in 1970. Since then, more and more researchers have reported the thermal characteristics of meridians and acupoints from different aspects. For example, Hu et al. [43, 44] observed the infrared radiant by an infrared imaging system and found that the tracks were consistent with the 14 meridians described by the ancient Chinese. Zhang et al. [45] measured the temperature 5 mm and 10 mm subcutaneous, respectively, along the meridians. They found that the high-temperature line along the meridians was formed after acupuncture, which was mainly emerged in the skin layer.

Furthermore, thermal characteristics may have a role in differentiating the syndromes. It was reported that thermal sensitivity measurement of meridians was used to differentiate the constitutions of yang deficiency, qi deficiency, and peace [46, 47].

### 2.4. Optical Characteristics

Studies addressing optical properties are relatively less and mainly focus on the high luminous properties and the transmission characteristics of light wave along the meridians. Yan et al. [48] found that there were 14 high luminous lines on the body surface, and the lines were significantly different from those located on both sides 5 mm away. Comparing the high luminous lines where 1934 points lie with the location of the 14 regular meridians on the body surface described by *Huang Di Nei Jin*, the rate of completely overlapped region was 92.97%, and the rate of basically consistent region was 6.72%. The results indicated that meridians and acupoints had high luminous biophysical properties. Liu et al. [49] designed an automatic measurement system to test the transmission characteristics of light wave along the meridians and found that there were significant differences between meridians and nonmeridians. Also, they found that the average attenuation factor of the pericardium meridian was lower than the spleen meridian.

### 2.5. Magnetic Characteristics

Magnetic characteristics of meridians and acupoints are still not fully defined. Li et al. [50] had examined meridians, acupoints, brain, and relative organs by using superconducting quantum interference device (SQUID) and functional magnetic resonance imaging (fMRI) in the national zero-magnetic laboratory located in China. They found a relatively stable circular current of electromagnetic and chemical oscillation along the low electric resistance pathway. Competition of different frequency oscillation often yielded resonance in some positions of the human body so as to form oscillatory network. Moreover, the electromagnetic and chemical oscillation circulation dominated the position of strange points in the body, which were possibly meridians and acupoints with regulative actions.

The human body is a magnetic field. Recently, the exploration of the relationship between meridians and resting-state brain networks by fMRI has gained popularity. Zhang et al. [51] used fMRI to investigate the specificity of acupuncture. By acupuncturing gallbladder (GB) 40, with kidney (KI) 3 as a control (belonging to the same nerve segment but different meridians), different brain areas were enhanced. It demonstrated that acupuncture at different acupoints could exert different modulatory effects on resting-state networks (RSNs).

Another interesting experiment was performed by Cao [52], who explored effects of acupoints magnetic stimulation on temperature field along meridians. In order to investigate the variation of temperature field before and after magnetic stimulation at acupoints, the infrared imaging temperature measurement was used to detect the distribution of temperature along the governor vessel on rabbit and human bodies directly. Compared with nonacupuncture points, the temperature of points on the governor vessel varied significantly. This showed that magnetic stimulation of acupoints could cause temperature variation along meridians.

### 2.6. Migration of Isotope along Meridians

Migration of isotope along channels was first discovered in 1950s. It is a relatively simply physical phenomenon, which follows physical laws. In 1980s, Meng and his group [53] in China had shown that slow movement of _ _^99m^TcO_4_^−^ along channels was observed after injecting the isotope into an acupoint, and the channels observed were similar to the meridians recorded in ancient Chinese literature. But they only proved that the channels had nothing to do with lymphatic network. Vessel and neural systems were not concerned.

In 2008, Zhang et al. [54] discovered low hydraulic resistance channels along meridians in minipigs. The low hydraulic resistance causes more fluid to flow along meridian lines compared with nonmeridian areas. The isotope tracing had been used to detect the stomach meridian in six minipigs. It was shown in two cases that, after injecting 0.1 mL _ _^99m^TcO_4_^−^ into one low hydraulic resistance point, a migration of isotope could be found along the meridian toward the other low hydraulic resistance point. Based on these findings, they deduced that the migration of isotope in the human body represented the interstitial fluid flow along low hydraulic resistance channels.

### 2.7. Myoelectric Activities

Myoelectricity is often used to study PSC in China. Zhu et al. [55] proved that PSC was closely related to the nerve-skeletal system. In their study, when large intestine (LI) 4 was stimulated, propagated sensation and myoelectricity along the large intestine meridian appeared. However, brachial plexus block anesthesia could eliminate this phenomenon. Another research in rats conducted by Ma et al. [56] also showed that the myoelectric activity of longissimus muscle was the foundation of PSC. These experiments show that meridians have the character of obvious myoelectric activity, and propagated sensation is closely related to myoelectricity.

## 3. Summary and Future Directions

Recent studies confirm that meridians and acupoints have many biophysical properties, which are different from those of nonacupuncture points. The characteristics include electric characteristics (i.e., high-electrical potential, conductance, and capacitance, low impedance and resistance), thermal characteristics (i.e., infrared radiant tracking along the meridians), acoustic characteristics (i.e., high guide sound with 2–15 Hz frequency, 0.5–10 mV amplitude, 6.2–10 cm/s bidirectional conduction velocity and being similar with sharp wave or sine wave), optical characteristics (i.e., high luminous properties and light wave spreading along the meridians), magnetic characteristics (i.e., a relative stable circular current of electromagnetic and chemical oscillation along the low electric resistance pathway), isotopic characteristics (i.e., migration of isotope along meridians), and myoelectric characteristics (i.e., obvious myoelectric activity). Therefore, the existence of meridians is scientifically supported.

These biophysical properties are the bases of PSC and further researches about morphology and acting pathways of meridians. They may also contribute to understanding the changes of body in different function states, diagnosing diseases, exploring new therapy, clarifying the pathogenesis, and differentiating symptomatic types and constitutions in TCM when every property is taken into consideration rather than using single property. Future basic studies should not only use a single method, such as electric, acoustic, optical, and magnetic method, to explore meridian essence, but also employ the methods of systemic biology, such as proteomics, genomics, transcriptomics, and other omics, to reveal the multitarget and multipath mechanisms of action. The basic studies should be further supported by clinical studies especially large randomized controlled trials. And appropriate diseases indicated for acupuncture intervention should be emphasized in this kind of investigations to show the therapeutic benefit.

## Figures and Tables

**Table 1:** Analysis of electrical skin resistance measurements (ESRMs) at acupoints in healthy humans.

ESR properties	Results of ESRMs
ESR (no significant difference)	397 (62.8%)
ESR↓	163 (25.9%)
ESR↑	71 (11.3%)

**Table 2:** The relationship between five different meridians and five musical tones cited in *Huang Di Nei Jin. *

Meridian	The tone name	Western name of the tone	Frequency for tone
Spleen	Gung	Do (C)	261.6 Hz
Lung	Sang	Re (D)	293.7 Hz
Liver	Gak	Mi (E)	329.6 Hz
Heart	Chi	Sol (G)	392.0 Hz
Kidney	Wu	La (A)	440.0 HZ
